# Obesity as a Risk Factor for Autoimmune Diseases: A Systematic Review and Meta‐Analysis

**DOI:** 10.1002/oby.70044

**Published:** 2025-11-04

**Authors:** Ilaria Spatocco, Giorgia Mele, Giusy De Rosa, Clorinda Fusco, Kristyna Ruggiero, Valeria Pellegrini, Francesca Carreras, Rosalba La Grotta, Antonio Ceriello, Claudio Procaccini, Giuseppe Matarese, Francesco Prattichizzo, Paola de Candia

**Affiliations:** ^1^ Dipartimento di Medicina Molecolare e Biotecnologie Mediche Università Degli Studi di Napoli Federico II Napoli Italy; ^2^ Laboratorio di Immunologia, Istituto Degli Endotipi in Oncologia, Metabolismo e Immunologia ‘G. Salvatore’, Consiglio Nazionale Delle Ricerche (IEOMI‐CNR) Napoli Italy; ^3^ Azienda Ospedaliera Universitaria ‘Federico II’ Napoli Italy; ^4^ IRCCS MultiMedica Milano Italy; ^5^ Unità di Neuroimmunologia Fondazione Santa Lucia Roma Italy

**Keywords:** autoimmunity, body mass index, meta‐analysis, obesity

## Abstract

**Objective:**

Obesity is characterized by a proinflammatory condition contributing to poor outcomes, but its association with autoimmunity is inconclusive. To fill this gap in knowledge, we searched PubMed and Embase for studies analyzing the association between obesity and the prevalence and/or incidence of autoimmune diseases.

**Methods:**

Adjusted odds ratios (OR) or hazard ratios (HR) with 95% CI relating to the prevalence or incidence of autoimmune diseases in people with BMI > 30, compared to BMI < 25, were pooled using generic inverse variance and fixed effect models. Of 1,311 records, 26 (8 cross‐sectional and 18 longitudinal) studies were included in the meta‐analysis.

**Results:**

Obesity, compared with normal weight, was associated with increased prevalence of rheumatoid arthritis and psoriasis (OR = 1.11 [1.06, 1.16], *p* < 0.00001; OR = 1.35 [1.14, 1.59], *p* = 0.0004, respectively) and increased risk of developing rheumatoid arthritis (HR = 1.30 [1.15, 1.49], *p* < 0.0001), psoriasis (HR = 1.18 [1.16, 1.20], *p* < 0.00001), multiple sclerosis (HR = 1.49 [1.25, 1.77], *p* < 0.00001), and Crohn's/ulcerative colitis (HR = 1.35 [1.11, 1.65], *p* < 0.003). Obesity was also significantly associated with incidence of any autoimmune disease (HR = 1.41 [1.24, 1.62], *p* < 0.00001).

**Conclusions:**

Although definitive conclusions are still precluded for the single diseases, overall evidence supports obesity as a risk factor for autoimmunity.

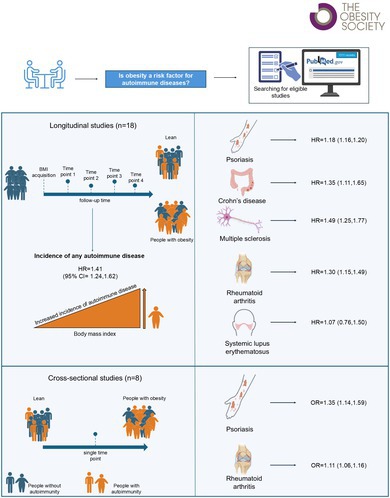

## Graphical abstract

Summary of the meta‐analysis including 26 studies (8 cross‐sectional, 18 longitudinal) investigating the association between obesity and autoimmune diseases. Findings show increased prevalence and incidence of rheumatoid arthritis, psoriasis, multiple sclerosis, and inflammatory bowel diseases in people with obesity, suggesting that obesity may influence immune function and increase autoimmune disease risk.

## 1 Introduction

The dramatic escalation of obesity, with a doubled worldwide prevalence since 1990, represents one of the most serious contemporary medical challenges [1, 2] (World Health Organization, 2024). Similarly, autoimmune diseases have experienced a dramatic increase in terms of both prevalence and incidence, especially in more affluent countries [3, 4]. Autoimmunity represents a heterogeneous group of diseases in terms of involved organs and tissues, but it is consistently characterized by an aberrant immune response, driven by autoreactive T cells, autoantibodies, and innate immune system dysregulation [5, 6]. Although the exact etiology remains unclear, autoimmune diseases are believed to result from a multifaceted interaction among genetic predisposition, concurring components such as sex, age, and previous viral infections, and other environmental factors, such as pollution, lifestyle, chronic stress, and smoking [7, 8, 9, 10, 11, 12, 13, 14, 15].

Mechanistic observations suggest that obesity may also increase the risk of developing an autoimmune disease through the induction of a chronic low‐grade proinflammatory status, which, being marked by an imbalance between the immune suppressive (i.e., regulatory T and B lymphocytes) and the proinflammatory (e.g., Th1 and Th17 cells) arms, creates the perfect milieu for the development of an aberrant immune response against self‐antigens [16, 17, 18, 19, 20, 21]. Moreover, obesity shares some key hallmarks with autoimmunity, such as dysregulated levels of cytokines and autoantibody production, as demonstrated in animal models and humans [22, 23, 24]. Several cohort studies actually suggest that obesity is associated with the prevalence or incidence of autoimmune diseases [25], which has led to hypothesizing a direct link between the immunological self‐tolerance, which suppresses self‐reactivity to avoid autoimmunity, and the nutrient‐ and energy‐sensing pathways dysregulated in people with obesity [26].

Nonetheless, any mechanistic hypothesis of a causal connection between obesity and autoimmunity still lacks strong clinical evidence; indeed, while obesity is nowadays fully recognized as a risk factor for diseases such as hypertension, type 2 diabetes, cardiovascular diseases, and cancer [27, 28], the same does not hold true for autoimmunity. In particular, a comprehensive meta‐analysis of the relevant epidemiological studies has never been performed, although it would provide tremendous relevance for human health. For this reason, we have here conducted a systematic review of the literature to collect all the available data linking obesity to any autoimmunity‐related endpoint and performed a quantitative meta‐analysis to directly explore its association with the prevalence and/or the incidence of autoimmune diseases.

## 2 Methods

### 2.1 Literature Search and Study Selection

We first screened PubMed, Prospero, and Cochrane databases to search for possible systematic reviews on the topic, either published or in preparation, finding no entries. Then, we searched relevant articles written only in English in Embase and PubMed with the following keywords: obesity, overweight, abdominal fat, body mass index (BMI), central adiposity, waist circumference, waist‐to‐hip ratio, autoimmunity, autoimmune, lupus, rheumatoid arthritis, multiple sclerosis, Graves' disease, Hashimoto's thyroiditis, psoriasis, Sjögren syndrome, coeliac disease, inflammatory bowel disease, myasthenia gravis, Addison's disease, scleroderma, vitiligo, alopecia areata, dermatomyositis, vasculitis, pernicious anemia, fibromyalgia. The search was limited to the temporal range 01/01/1945 to 31/03/2024. As an example of the search strategy, the strings used in PubMed are attached as online Supporting Information Appendix S1. We decided not to include type 1 diabetes (T1D) as one of the diseases of interest since body weight and adiposity are inherently linked to the etiopathogenesis of this condition also independently of immune‐related mechanisms [29]. In addition, given the usually very early onset of this disease, the population included in the studies would have been much younger than all the others. Thus, including T1D as an outcome could have introduced confounding factors, calling out for its exclusion from the study. We also decided not to include studies concerning people with multiple autoimmune diseases since we wanted to evaluate the link between obesity and autoimmunity in a clear 1‐to‐1 association, deriving results relevant for one specific autoimmune disease at a time. Four investigators (I.S., G. Mele, G.D.R., and C.F.) identified the eligible abstracts, and two of them (I.S. and G. Mele) independently reviewed the eligible abstracts to finalize the inclusion list. Eligibility criteria for all studies were: 1‐They report data relative to an association between BMI and any autoimmune disease prevalence or incidence, except for T1D; 2‐They provide clear indication of the population included, individuals' characteristics, and endpoints. Exclusion criteria were: 1‐Studies with unclear design, not reporting outcome estimates, or without available full text; 2‐Studies from where it was not possible to extrapolate stratified data to compare people with BMI > 30 with those with BMI < 25, e.g., providing estimates for the association between BMI and disease incidence/prevalence using only BMI as a continuous, and not categorical, variable or using measures of adiposity other than BMI; 3‐Studies concerning pediatric individuals; 4‐Studies evaluating individuals with multiple autoimmune diseases. The protocol is registered in Prospero with the accession number CRD42024529967. We followed the Meta‐analysis of Observational Studies in Epidemiology (MOOSE) checklist to conduct this systematic review (reported in the online Supporting Information).

### 2.2 Data Extraction and Quality Assessment of the Studies

All relevant data from the included studies were extracted by two independent reviewers (I.S. and G. Mele) using a prespecified, standardized data extraction template in Microsoft Excel (Microsoft, Seattle, WA). The collected data are presented in Table 1. In cases of disagreement between the reviewers, the differences were resolved through consultation with two senior team members (F.P. and P.d.C.). Data were then checked for accuracy by another investigator (C.P.). Collected information was study type, number of patients in each group, age and sex of the enrolled patients, and severity of the disease. No masking procedure was adopted for either study selection or data extraction. Three authors (V.P., F.C., and R.L.G.) independently assessed the quality of included studies using the Risk Of Bias In Non‐randomized Studies‐of Interventions (ROBINS‐I) tool. Discrepancies regarding the evaluation of selected items were resolved by a fourth author (F.P.).

**TABLE 1:** Main characteristics of the 26 included studies, testing the association between obesity and either the prevalence or the incidence of the indicated autoimmune diseases.

	Study type	Publication doi	Publication title	Sample size (Total)	Sample size (Women)	Sample size (Men)	Age, mean (± SD or IQR), years	Length of follow‐up (years)
Rheumatoid arthritis								
Althumiri N.A. et al. (2021)	Cross‐sectional	10.3390/healthcare9030311	Obesity in Saudi Arabia in 2020: Prevalence, distribution, and its current association with various health conditions	267	—	—	54	—
Dar L. et al. (2018)	Cross‐sectional	10.1111/ijcp.13045	Are obesity and rheumatoid arthritis interrelated?	11,406	8822	2584	62.2	—
Hedström A.K. et al. (2019)	Cross‐sectional	doi.org/10.1136/rmdopen‐2018‐000856	Interplay between obesity and smoking with regard to RA risk	3572	2547	1025	52.1	—
Symmons D.P. et al. (1997)	Cross‐sectional	10.1002/art.1780401106	Blood transfusion, smoking, and obesity as risk factors for the development of rheumatoid arthritis: results from a primary care‐based incident case–control study in Norfolk	90	—	—	44.5	—
De Hair J.H. et al. (2013)	Longitudinal	10.1136/annrheumdis‐2012‐202,254	Smoking and overweight determine the likelihood of developing rheumatoid arthritis	15	9	6	47 (8)	1.08
Linauskas A. et al. (2019)	Longitudinal	10.1002/acr.23694	Body fat percentage, waist circumference, and obesity as risk factors for rheumatoid arthritis: a Danish cohort study	666	456	210	57 (53–61)	20.1
Ljung L. et al. (2016)	Longitudinal	10.1186/s13075‐016‐1171‐2	Abdominal obesity, gender and the risk of rheumatoid arthritis—a nested case–control study	554	379	178	51.9 (9.1)	27
Lu B. et al. (2014)	Longitudinal	10.1136/annrheumdis‐2014‐205,459	Being overweight or obese and risk of developing rheumatoid arthritis among women: a prospective cohort study	1181	1181	0	—	Cohort 1: 32 Cohort 2: 20
Marchand N.E. et al. (2021)	Longitudinal	10.3899/jrheum.200056	Abdominal obesity in comparison with general obesity and risk of developing rheumatoid arthritis in women	844	—	—	47,8	28
Turesson C. et al. (2016)	Longitudinal	10.1093/rheumatology/kev313	A high body mass index is associated with reduced risk of rheumatoid arthritis in men, but not in women	462	275	187	52,55	Cohort 1: 5 Cohort 2: 18
Psoriasis								
McGowan J.W. et al. (2005)	Cross‐sectional	10.1001/archderm.141.12.1601	The skinny on psoriasis and obesity	400	0	400	—	—
Naldi L. et al. (2005)	Cross‐sectional	10.1111/j.0022‐202X.2005.23681.x	Cigarette smoking, body mass index, and stressful life events as risk factors for psoriasis: results from an Italian case–control study	550	—	—	—	—
Smith B. et al. (2023)	Cross‐sectional	10.12788/cutis.0807	Association between psoriasis and obesity among US adults in the 2009–2014 National Health and Nutrition Examination Survey	—	—	—	—	—
Zhang C. et al. (2011)	Cross‐sectional	10.1111/j.1468‐3083.2010.03706.x	The effect of overweight and obesity on psoriasis patients in Chinese Han population: a hospital‐based study	4452	1891	2561	36.54 (±13.08)	—
Han J.H. et al. (2019)	Longitudinal	doi.org/10.1111/1346‐8138.14939	Increased risk of psoriasis in subjects with abdominal obesity: A nationwide population‐based study	399,461	181,770	217,691	50.46	5.32
Kumar S. et al. (2013)	Longitudinal	10.1111/jdv.12001	Obesity, waist circumference, weight change and the risk of psoriasis in US women	809	809	0	62	12
Norden A. et al. (2022)	Longitudinal	10.1016/j.jaad.2021.06.012	Risk of psoriasis according to body mass index: A retrospective cohort analysis	1,506,547	870,841	689,706	47, 52	11
Setty A.R. et al. (2007)	Longitudinal	10.1001/archinte.167.15.1670	Obesity, waist circumference, weight change, and the risk of psoriasis in women: Nurses' Health Study II	880	880	0	36, 02	14
Snekvik I. et al. (2017)	Longitudinal	10.1016/j.jid.2017.07.822	Obesity, waist circumference, weight change, and risk of incident psoriasis: prospective data from the HUNT study	369	185	184	47.0 (13.3)	13
Multiple sclerosis								
Høglund R.A.A. et al. (2021)	Longitudinal	10.1212/WNL.0000000000012957	Association of body mass index in adolescence and young adulthood and long‐term risk of multiple sclerosis: a population‐based study	1409	—	—	24	12
Xu Y. et al. (2021)	Longitudinal	10.1177/1352458520928061	Higher body mass index at ages 16 to 20 years is associated with increased risk of a multiple sclerosis diagnosis in subsequent adulthood among men	952	0	952	—	—
Crohn's disease and ulcerative colitis								
Chan S.M. et al. (2013)	Longitudinal	10.1038/ajg.2012.453	Body mass index and the risk for Crohn's disease and ulcerative colitis: data from a European Prospective Cohort Study (The IBD in EPIC Study)	75	48	27	56.4	8
Chan S.S.M. et al. (2022)	Longitudinal	10.1016/j.cgh.2021.06.049	Obesity is associated with increased risk of Crohn's disease, but not ulcerative colitis: A pooled analysis of five prospective cohort studies	563	—	—	—	16
Khalili H. et al. (2015)	Longitudinal	10.1097/MIB.0000000000000283	Measures of obesity and risk of Crohn's disease and ulcerative colitis	153	153	0	35.09	20
Systemic lupus erythematosus								
Cozier Y.C. et al. (2019)	Longitudinal	10.1016/j.semarthrit.2018.10.004	A prospective study of obesity and risk of systemic lupus erythematosus (SLE) among Black women	127	127	0	43	20
Tedeschi S.K. et al. (2017)	Longitudinal	10.1016/j.semarthrit.2017.05.011	Obesity and the risk of systemic lupus erythematosus among women in the Nurses' Health Studies	268	—	—	42.5	Cohort 1: 36 Cohort: 24

### 2.3 Statistical Analysis

Upon collection of the published studies to be analyzed, we grouped the studies based on the specific autoimmune disease and then further on the type of study (either longitudinal or cross‐sectional). For the quantitative meta‐analysis, we used the generic inverse variance method to explore the effect of living with obesity, compared with normal weight (i.e., BMI > 30 versus BMI < 25), on autoimmune disease incidence or prevalence, starting from either collected odds ratios (ORs) or hazard ratios (HRs) with the relative confidence intervals (CIs). We opted to perform only this comparison to isolate the effect of established or “extreme” phenotypes, either pathological or healthy. Indeed, people in the overweight range, that is, those with BMI between 25 and 30, are historically characterized by both beneficial and unfavorable outcomes when compared to lean people, with a highly context‐ and study design‐dependent effect [30]. In detail, ORs were used for cross‐sectional studies and assessed the prevalence of autoimmune diseases in lean individuals and people with obesity, providing a measure of the strength of association between the two variables (obesity and autoimmunity), with 1 for no association, above 1 for a positive, and below 1 for a negative association, without considering the time parameter. Differently, HRs were used for longitudinal studies on disease incidence and compared the hazards of developing an autoimmune disease over time in lean individuals and people with obesity, with 1 for no divergence, and HRs different than 1 showing that the onset of autoimmunity is not occurring at an equal rate in the two compared groups. For those studies reporting HRs or ORs for multiple BMI strata, we calculated the relative HRs or ORs with 95% CIs by pooling adjusted data from all the strata above BMI of 30 through generic inverse variance, once it was established that the comparator group was BMI < 25 in all cases. These cutoff points were adapted for studies with non‐Caucasian individuals, for example, Chinese, tailoring the analysis according to the recommended cutoff to identify obesity in these groups [31]. Given the small number of items included in each comparison, we opted to use the fixed effect model [32]. Statistical heterogeneity between studies was evaluated by the *I* ^2^ statistic. After pooling data for individual diseases, we also performed an overall meta‐analysis pooling data relative to the longitudinal association between BMI > 30 versus BMI < 25 and the incidence of any autoimmune disease. For this analysis (the only one with more than 10 included studies) we used the random effect model and CIs were calculated with the Hartung‐Knapp‐Sidik‐Jonkman method while tau^2^ was calculated with the DerSimonian and Laird method. For this overall comparison, two sensitivity analyses were performed, one based on a mechanistic assumption and the other based on the publication bias hypothesis. In the first case, studies reporting data on the longitudinal incidence of systemic lupus erythematosus (SLE) were excluded, since this disease is different from the others in terms of etiopathogenesis. Indeed, SLE is held to be a type III hypersensitivity response with potential type II involvement [33]. In the second case, a funnel plot was generated and the six studies exceeding the 95% CI of the plot were excluded from the analysis [34]. All the analyses were performed using the online software Review Manager 5.4 (Cochrane Collaboration, London, UK) with the exception of the funnel plot, which was generated with the online tool https://metaanalysisonline.com/.

## 3 Results

### 3.1 Included Studies and Resulting Populations

**FIGURE 1:**
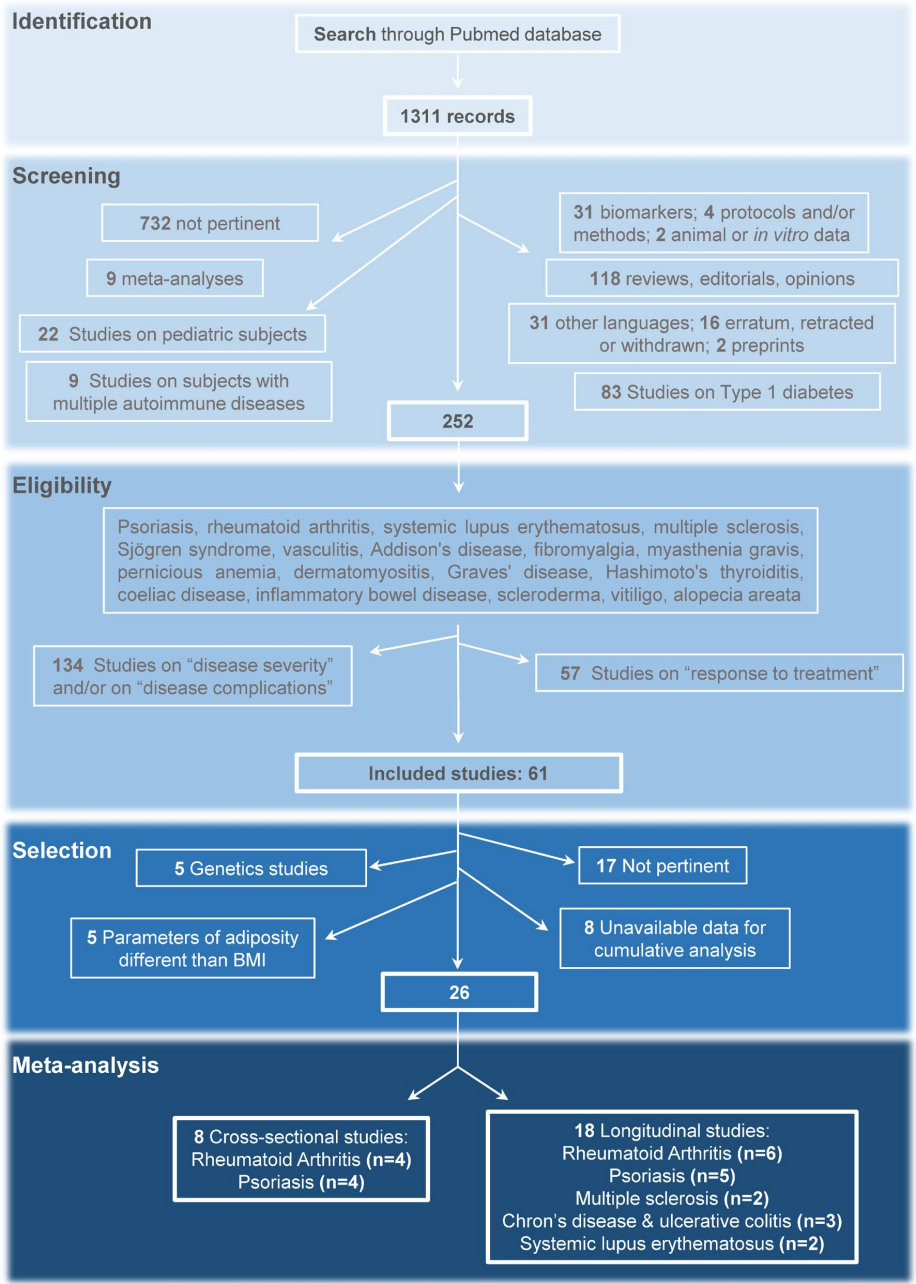
Flowchart for the excluded and included studies. [Color figure can be viewed at wileyonlinelibrary.com]

Upon identifying 1311 pertinent manuscripts to be evaluated, 1059 were immediately excluded since they were: not pertinent (732); meta‐analyses, reviews, editorials, or opinions (127); preprints, errata, retracted, withdrawn, or written in languages other than English (49); reporting animal or in vitro data, protocols and/or methods, or focused on biomarker identification (37); manuscripts pertaining to T1D (83); manuscripts concerning individuals affected by multiple autoimmune diseases (9); and manuscripts about autoimmune diseases affecting pediatric patients (due to irreconcilable differences in disease initiation and progression compared to adults) (22) (Figure 1). The remaining 252 studies covered four different clinical subjects: “autoimmune disease status or onset” (*n* = 61), “autoimmune disease severity” and/or “autoimmunity‐related complications” (*n* = 134), and “response to treatment for autoimmunity” (*n* = 57). Of the 61 studies analyzing autoimmunity per se, 35 studies could not be utilized for the final analysis due to several reasons. In particular, after a more thorough screening, we found that 17 studies were not pertinent to the specific topic of our meta‐analysis, 5 studies used parameters different from BMI for assessing adiposity, such as waist circumference or silhouette size, 5 studies performed genetic analyses (e.g., GWAS, polymorphism studies), and 8 studies were excluded due to the absence of other studies within the same category (i.e., same pathology in the same study type categories), insufficient information, or the use of a different analytical approach, which precluded the possibility of pooling the data (Figure 1 and Table S1).

Finally, the other 26 articles were selected as suitable for the meta‐analysis (Figure S1); of those, 8 articles reported cross‐sectional studies exploring the relationship between BMI and the prevalence of rheumatoid arthritis (RA) (*n* = 4) and psoriasis (*n* = 4) (the study main characteristics are summarized in Table 1). Overall, the 8 studies reported data from 20,737 people (31,68% of whom were males), with a mean age of 48.1 years. The other 18/26 articles reported longitudinal studies exploring the association of baseline BMI with the subsequent incidence of autoimmune diseases, with a mean follow‐up of 16.63 years. Those studies explored the relationship between obesity and the incidence of RA (*n* = 6), psoriasis (*n* = 5), multiple sclerosis (MS) (*n* = 2), Crohn's/ulcerative diseases (*n* = 3), and SLE (*n* = 2) (the study main characteristics are summarized in Table 1). Overall, the 18 studies reported data from 1,915,335 people (47,46% of whom were males), with a mean age of 41.8 years. The risk of bias summary is reported in Figure S2. In brief, of the 8 cross‐sectional studies, 5 were at high risk of bias while the other 4 were at moderate risk; of the 18 longitudinal studies, 3 were at low risk of bias, 4 were at high risk, and the remaining 11 were at moderate risk, with the most common issue being the lack of accurate description of the methodology used to manage missing data.

### 3.2 Obesity and Prevalence or Incidence of Rheumatoid Arthritis

**FIGURE 2:**
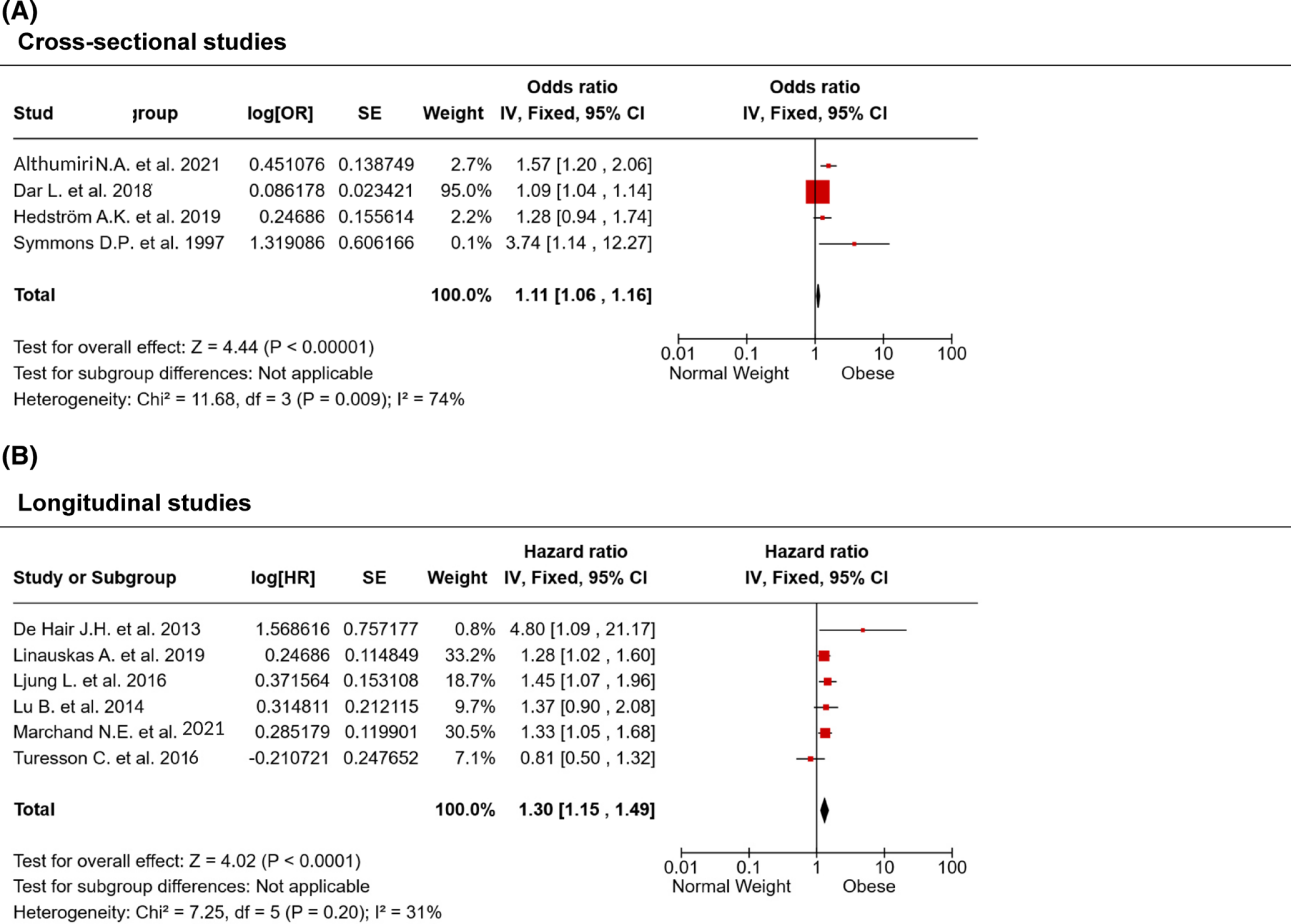
Obesity and rheumatoid arthritis. Forest plots summarizing the (A) prevalence and the (B) incidence of rheumatoid arthritis in people with obesity compared with normal weight. Overall estimates are calculated using the generic inverse variance method. [Color figure can be viewed at wileyonlinelibrary.com]

Four selected studies report a cross‐sectional evaluation of rheumatoid arthritis (RA) prevalence in populations living in England, Sweden, Saudi Arabia, and Israel with either obesity or not [35, 36, 37, 38]. Obesity was associated with a slight but highly significant increase in odds of having RA compared with normal weight (OR = 1.11, CI 1.06–1.16, *p* < 0.00001), with a high heterogeneity across studies (*I* ^2^ = 74%, *p* = 0.009) (Figure 2A).

Six studies [39, 40, 41, 42, 43, 44] reported longitudinal evaluations of RA incidence in Caucasian populations. Our analysis demonstrated that obesity was associated with a 30% increased risk of developing RA compared to normal weight (HR = 1.30, CI 1.15–1.49, *p* < 0.0001) (Figure 2B). The studies were found to be moderately heterogeneous (*I* ^2^ = 31%, *p* = 0.20).

### 3.3 Obesity and Prevalence or Incidence of Psoriasis

**FIGURE 3:**
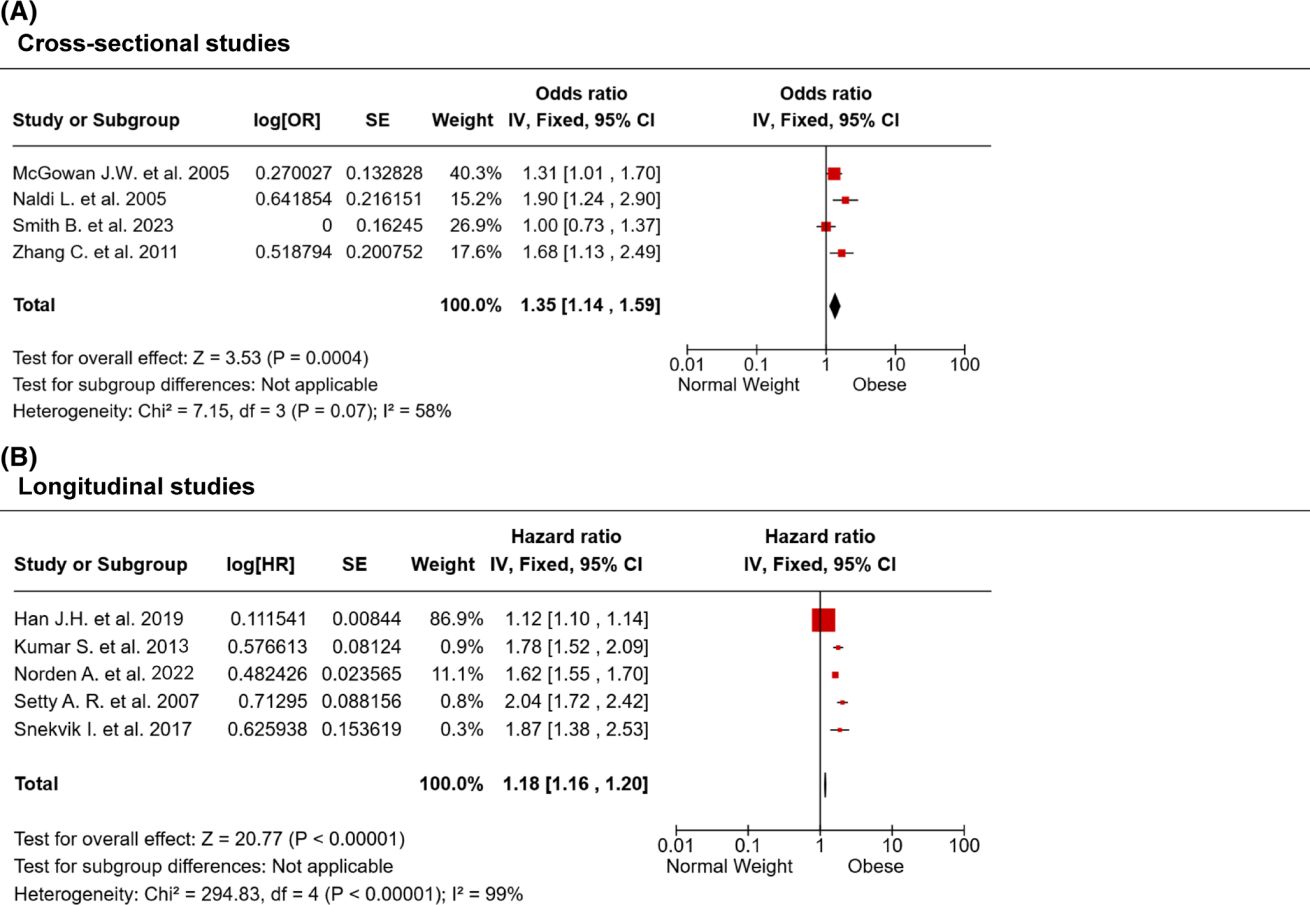
Obesity and psoriasis. Forest plots summarizing the (A) prevalence and the (B) incidence of psoriasis in people with obesity compared with normal weight. Overall estimates are calculated using the generic inverse variance method. [Color figure can be viewed at wileyonlinelibrary.com]

Four selected studies report a cross‐sectional evaluation of the prevalence of psoriasis in Chinese, American, and Italian people with either obesity or not [45, 46, 47, 48]. Our analysis showed that obesity was associated with a 35% increased odds of the outcome (psoriasis) compared to normal weight (OR = 1.35, CI 1.14–1.59, *p* = 0.0004) (Figure 3A). The studies were moderately heterogeneous (*I* ^2^ = 58%, *p* = 0.07).

Additionally, five studies performed a longitudinal evaluation of psoriasis incidence in American, Asian, and European populations [49, 50, 51, 52, 53]. Our analysis showed that obesity was associated with a slight (but statistically significant) 18% increased risk of developing psoriasis compared to normal weight (HR = 1.18, CI 1.16–1.20, *p* = 0.00001) (Figure 3B). The studies showed a very high grade of heterogeneity (*I* ^2^ = 99%, *p* < 0.00001).

### 3.4 Obesity and Incidence of Multiple Sclerosis, Crohn's Disease, and Systemic Lupus Erythematosus

#### 3.4.1 Multiple Sclerosis

**FIGURE 4:**
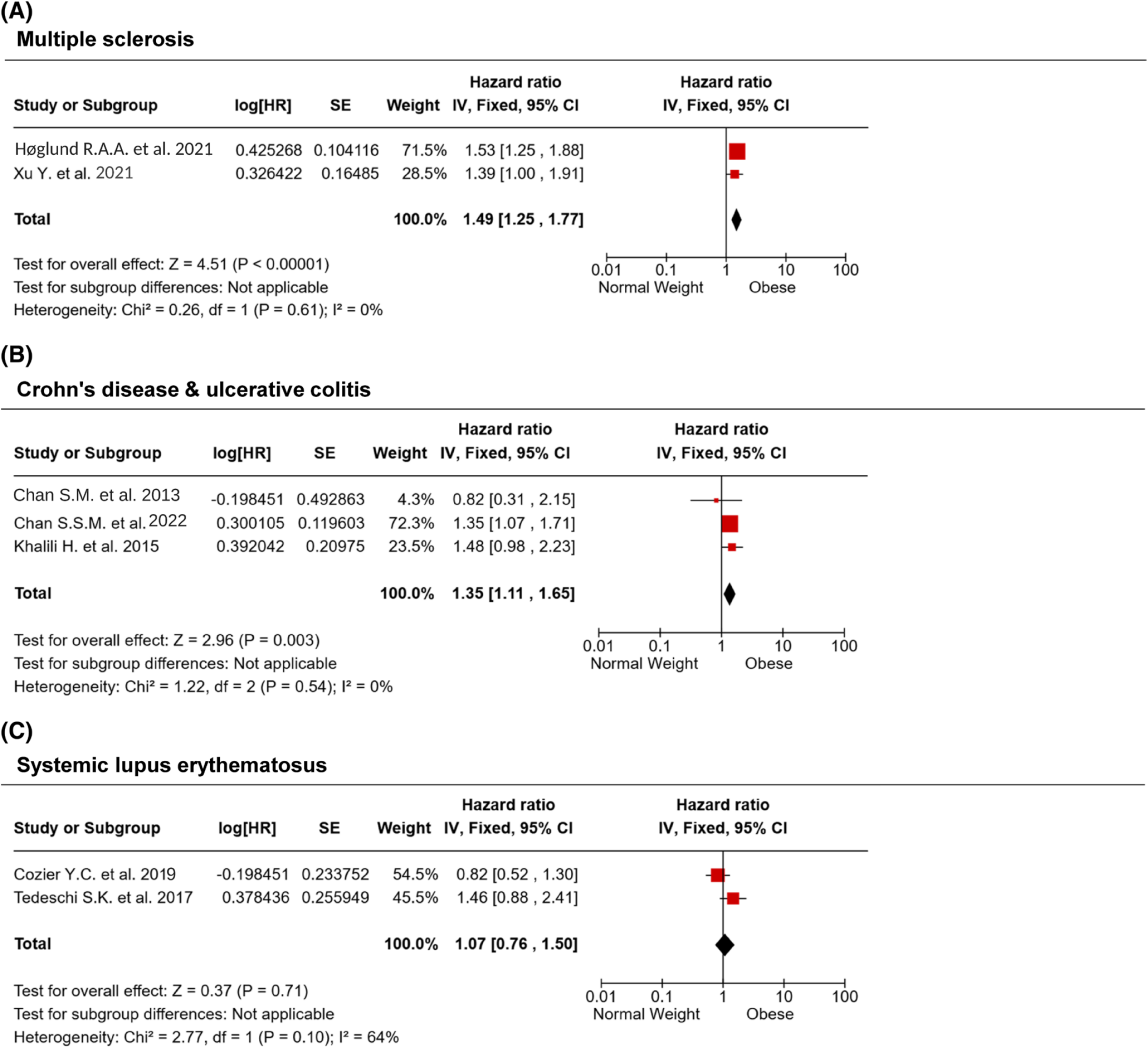
Obesity and other autoimmune diseases. Forest plots summarizing the incidence of (A) multiple sclerosis, (B) Crohn's disease and ulcerative colitis, and (C) systemic lupus erythematosus in people with obesity compared with normal weight. Overall estimates are calculated using the generic inverse variance method. [Color figure can be viewed at wileyonlinelibrary.com]

Two selected studies performed a longitudinal evaluation of multiple sclerosis (MS) incidence, performed on Norwegian and Swedish populations [54, 55]. Our analysis showed that obesity is associated with a 49% increased risk of developing MS, compared to normal weight (HR = 1.49, CI 1.25–1.77, *p* < 0.00001) (Figure 4A). The studies demonstrated an insignificant heterogeneity (*I* ^2^ = 0%, *p* = 0.61).

Despite including a large population (302,043 participants), we were unable to consider the work of Munger K.L. et al. [56] in our research, as we excluded studies involving pediatric patients. This exclusion was necessary due to the significant differences in terms of the development of autoimmune diseases, such as MS [57], when they occur at an early age compared to their manifestation later in life.

#### 3.4.2 Crohn's Disease

Three selected studies performed a longitudinal evaluation of Crohn's disease incidence in American and European people with either obesity or not [58, 59, 60]. Our analysis showed that obesity was associated with a 35% increased risk of developing the disease compared to normal weight (HR = 1.35, CI 1.11–1.65, *p* = 0.003) (Figure 4B). The studies showed insignificant heterogeneity (*I* ^2^ = 0%, *p* = 0.54).

#### 3.4.3 Systemic Lupus Erythematosus

Two studies performed a longitudinal evaluation of systemic lupus erythematosus (SLE) incidence in American populations [61, 62]. Since the two studies found conflicting results in regard to the risk of developing SLE in people with obesity compared to normal weight, our analysis did not find any significant overall effect (HR = 1.07, CI 0.76–1.50, *p* = 0.71) (Figure 4C). The studies showed relevant heterogeneity (*I* ^2^ = 64%, *p* = 0.10).

### 3.5 Obesity and the Longitudinal Association With Any Autoimmune Disease

**FIGURE 5:**
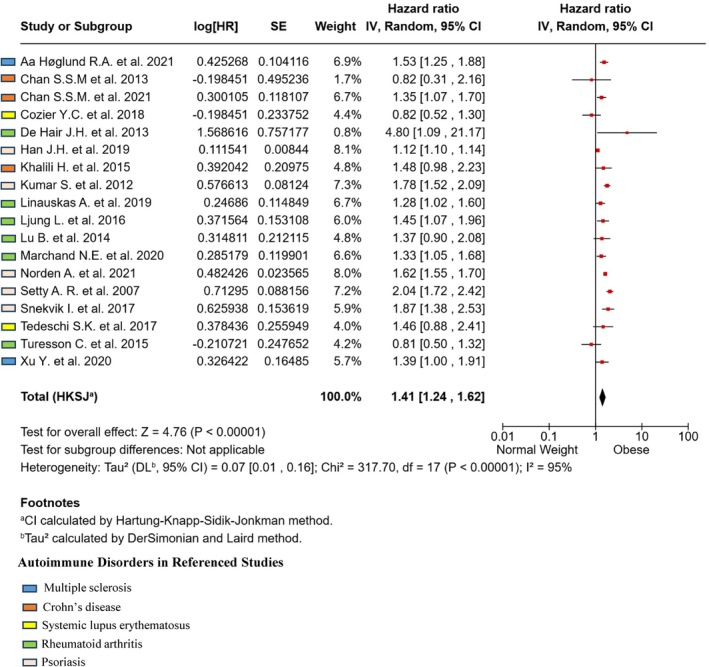
Obesity and the incidence of any autoimmune disease. Forest plot summarizing the incidence of all autoimmune diseases included in the study (each disease recognizable by the indicated color code) in people with obesity compared with normal weight. Overall estimate is calculated using the generic inverse variance method.

To explore whether obesity represents a risk factor for autoimmunity as a whole, we also performed an overall comparison of the incidence of any autoimmune disease in people with obesity compared with normal weight, taking advantage of all the longitudinal studies collected. Obesity was associated with an increased risk of any autoimmune disease (HR = 1.41, CI 1.24–1.62, *p* < 0.00001), with high heterogeneity across studies (*I* ^2^ = 95%, *p* < 0.00001) (Figure 5).

We then performed two sensitivity analyses. First, given that SLE has a different etiopathogenesis compared with the other four diseases collected [63], we excluded the two studies dealing with this disease and found that the overall result slightly increased (HR = 1.45, CI 1.26–1.66, *p* < 0.00001; heterogeneity *I* ^2^ = 95%, *p* < 0.00001, data not shown). After having evaluated the risk of bias (funnel plot, Figure S3), a sensitivity analysis performed by excluding the six points falling outside the 95% CI still provided significant results, with no heterogeneity (HR = 1.42, CI 1.27–1.58, *p* < 0.00001; heterogeneity *I* ^2^ = 0%, *p* < 0.51, data not shown).

## 4 Discussion

A substantial body of epidemiological data suggests that obesity could represent a risk factor for the development of autoimmune diseases, but, to our knowledge, this is the first meta‐analysis summarizing data from all studies that concern this risk. Even though the number of studies is too small to draw definitive conclusions relative to each autoimmune disease, we believe that the analysis has provided compelling evidence relative to the association between obesity and autoimmunity as a whole.

In the effort of maintaining a comprehensive analytical goal, we had to make several choices to synthesize data in a meaningful manner. For instance, we excluded studies dealing with pediatric populations, given that this would have introduced an excessive heterogeneity in terms of the analyzed population. We also opted not to include T1D as an outcome to be collected, given the well‐documented evidence that excessive adiposity modulates the risk of T1D from the very beginning of life, while additional features of hyperglycemia are obviously intertwined with obesity [64, 65], which would have introduced a bias in the results. In addition, we decided to focus only on a single comparison, that is, people with BMI > 30 versus those with BMI < 25, to ensure that the results would not be confounded or diluted by the effect of intermediate overweight. On the other hand, results aggregated in this manner lack granularity and impede the discernment of a potential escalating association or, alternately, of a nonmonotonic relationship. Furthermore, the use of BMI < 25 as the reference group was a choice forced by the design of all the included studies, although it might potentially hold a bias relative to the inclusion of underweight people. However, we reasoned that this choice unlikely represents a key issue for two reasons: (i) the possible effects on the results should, at most, dilute the pathological association between obesity and autoimmune diseases, rather than promoting it; (ii) the number of individuals with eating disorders included in observational studies is likely to be very small, with the exception of register‐based studies, which were a minority portion of those covered here.

It should also be emphasized here that observational studies are inherently linked to the risk of residual confounders and thus suffer from the limitations of all similar analyses, i.e., being incapable of providing evidence of causality. An additional implication of such an approach is that different populations in terms of age and other characteristics (e.g., demographic attributes) might have been considered together. Thus, the results relative to the overall association of obesity with autoimmunity should be interpreted with caution. Furthermore, given the heterogeneity in terms of study design, we did not perform stratification or subgroup analyses according to the baseline characteristics of the studies. For instance, we did not stratify the analyses according to the length of follow‐up, considering together studies with shorter and longer follow‐ups. Also, we used HRs to pool data and not the number of events collected in each study. These aspects impede exploring whether the increased risk can be attributed to a faster rate of development of the condition in people with obesity or whether there is an overall increased numerical incidence of autoimmune diseases. Finally, studies with long follow‐ups might be possibly affected by the risk of misclassification since body weight may fluctuate over time.

Notwithstanding these limitations, the overall trend observed across studies demonstrates a consistent association between obesity and an elevated risk of developing autoimmune disorders. In particular, this systematic review presents a broad generalizability aspect, since it encompasses studies spanning different time periods, geographical regions, and populations and covers multiple autoimmune diseases; moreover, the inclusion of both cross‐sectional and longitudinal studies enhances the generalizability of the study by capturing both prevalence and incidence data. These data substantiate a plethora of mechanistic studies linking overnutrition to immune dysregulation and provide the foundation to further deepen the studies in this complex field. Our findings might suggest that obesity does not affect all autoimmune disorders in the same way: indeed, we found a positive association between obesity and the prevalence or incidence of all the autoimmune diseases tested, except SLE. The small number of SLE studies might eventually explain this observation. However, a mechanistic possibility could also be conceived: while RA, psoriasis, and MS are consistently characterized by an altered activation of the T cell compartment, SLE is now recognized to be mostly driven by the aberrant expansion and activation of autoreactive B cells [66], which may suggest different intermingling with obesity‐driven inflammation. On the other hand, the actual “autoimmune” pathogenesis of some of the diseases we have here evaluated (such as psoriasis and bowel hyperinflammatory conditions) is still not completely characterized, with self‐antigens remaining elusive. Hence, we do acknowledge that our work is not able to fully consider the pathogenetic complexity of each disease and that further analyses will have to disentangle the association possibly existing between obesity and immune‐mediated diseases overall.

Finally, factors such as diet, physical activity, genetic predisposition, and socioeconomic status, which may contribute to both obesity and autoimmune disease susceptibility, are not uniformly accounted for across the range of studies, potentially affecting the strength of the results. Indeed, the association between obesity and autoimmune diseases appears more prominent in affluent countries (i.e., North America and Europe), likely due to a combination of higher obesity rates, dietary patterns, and environmental influences that align with Western lifestyle. However, as obesity rates are rising in developing nations, a global shift in autoimmune disease burden may occur, necessitating further research into region‐specific risk factors [4].

## 5 Conclusion

The results of this meta‐analysis envisage clear clinical implications. Beyond the existing knowledge prompting a widespread adoption of programs of obesity prevention and management in the general population, our data suggest that individuals with obesity should be perceived as living with an increased risk of autoimmune diseases, an observation with public health implications, providing an additional scientific rationale to encourage obesity management and prevention at both the population and the clinical level.

## Author Contributions

P.d.C. conceived the idea and F.P. prepared the draft of the protocol. P.d.C. and F.P. contributed to study design and data interpretation. P.d.C., F.P., I.S., and G. Mele wrote the manuscript. I.S., G. Mele, G.D.R., K.R. and C.F. performed the data collection and selection and participated in the manuscript preparation. I.S., G. Mele, and G.D.R. checked the accuracy of the collected information and prepared the figures. R.L.G., F.C., and V.P. assessed the quality and risk of bias of the included studies and helped with the preparation of the tables. A.C., C.P., and G. Matarese reviewed the manuscript for intellectual and methodological content. All authors approved the final version and agreed to be accountable for all aspects of the work.

## Conflicts of Interest

The authors declare no conflicts of interest.

## Supporting information


**Figure S1:** Main characteristics of the included studies.
**Figure S2:** Risk of publication bias.
**Figure S3:** oby70044‐sup‐0001‐Figures.pptx.

**Table S1:** References of the 35 studies potentially useful for testing the association between obesity and autoimmune diseases, and then excluded for the reasons indicated in the last column on the right (see also Figure 1).

## Data Availability

The data that support the findings of this study are available on request from the corresponding author. The data are not publicly available due to privacy or ethical restrictions.
