# Supplementary material for: Obesity as a Risk Factor for Autoimmune Diseases: A Systematic Review and Meta‐Analysis
Source: Obesity (Silver Spring). 2025 Nov 4;34(1):36–50. doi: 10.1002/oby.70044 (PMC12724045; doi:10.1002/oby.70044)
Supplement: Supplementary file 1 — Figure S1: Main characteristics of the included studies. Figure S2: Risk of publication bias. Figure S3: oby70044‐sup‐0001‐Figures.pptx. [file OBY-34-36-s002.pptx]

## Slide 1
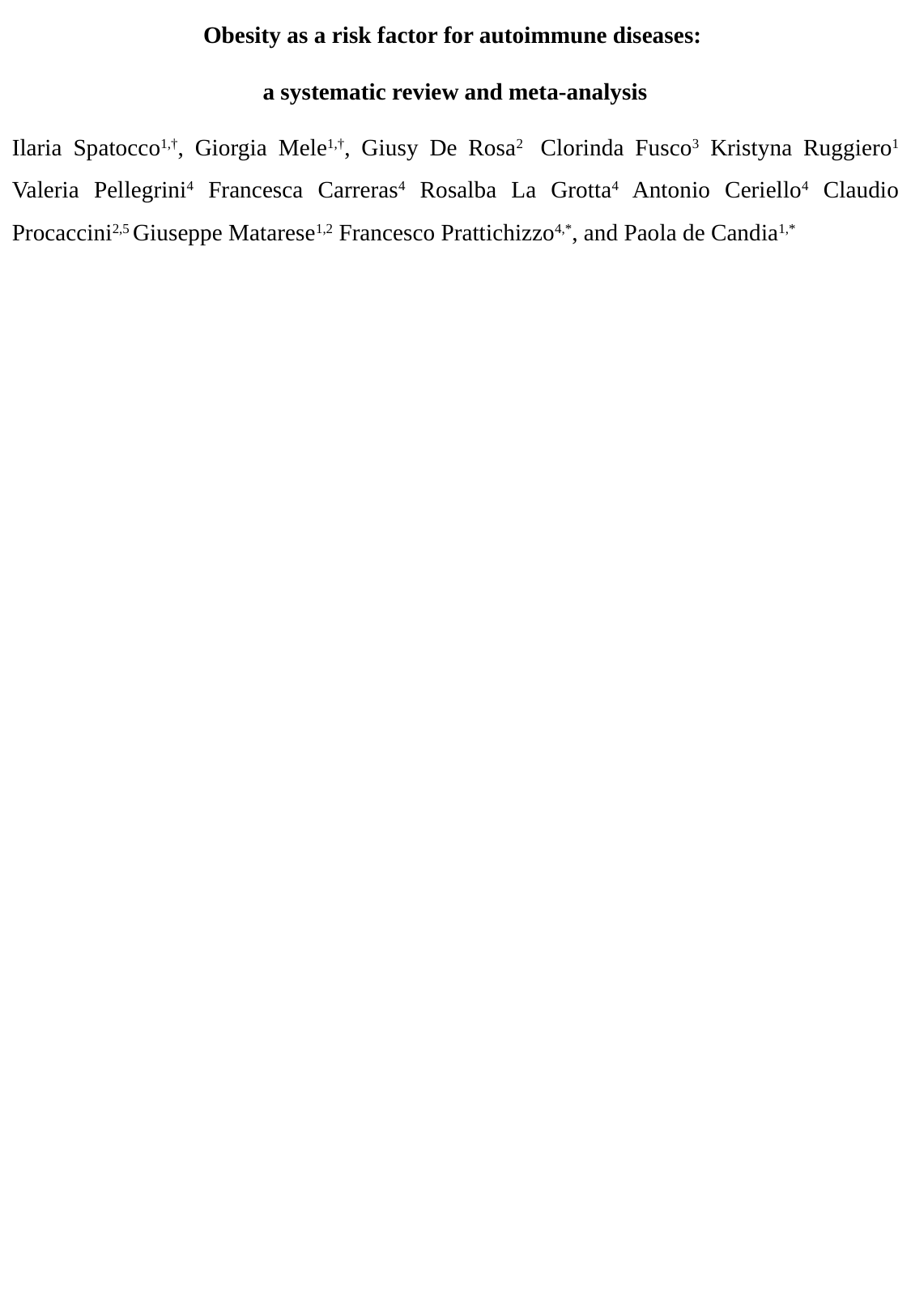

Obesity as a risk factor for autoimmune diseases:
a systematic review and meta-analysis
Ilaria Spatocco1,†, Giorgia Mele1,†, Giusy De Rosa2 Clorinda Fusco3 Kristyna Ruggiero1 Valeria Pellegrini4 Francesca Carreras4 Rosalba La Grotta4 Antonio Ceriello4 Claudio Procaccini2,5 Giuseppe Matarese1,2 Francesco Prattichizzo4,*, and Paola de Candia1,*

## Slide 2
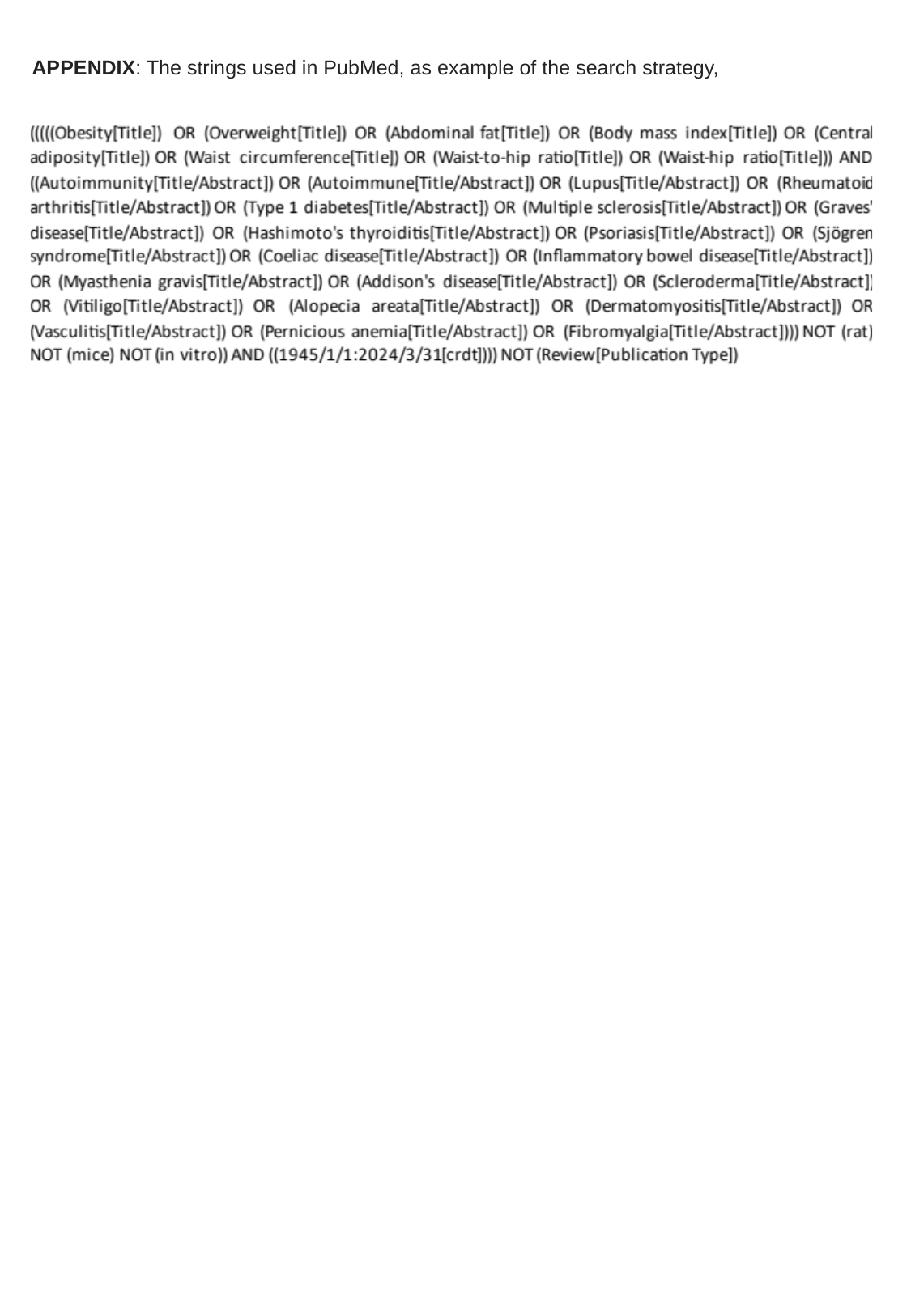

APPENDIX: The strings used in PubMed, as example of the search strategy,

## Slide 3
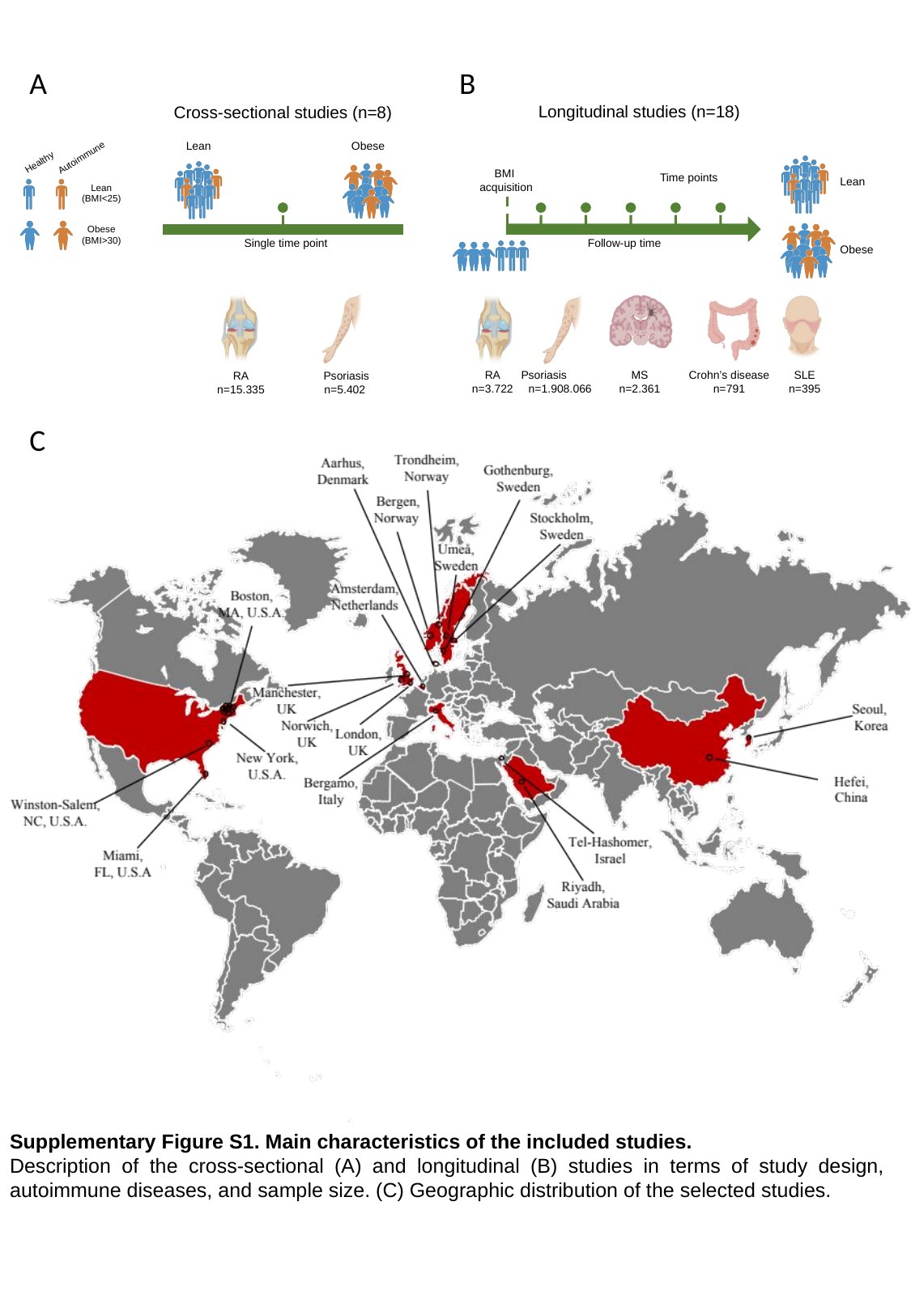

A
B
Longitudinal studies (n=18)
Cross-sectional studies (n=8)
Obese
Lean
Autoimmune
Healthy
BMI
acquisition
Time points
Lean
Lean (BMI<25)
Obese
(BMI>30)
Single time point
Follow-up time
Obese
RA
n=3.722
Psoriasis n=1.908.066
MS
n=2.361
Crohn’s disease
n=791
SLE
n=395
RA
n=15.335
Psoriasis
n=5.402
C
Supplementary Figure S1. Main characteristics of the included studies.
Description of the cross-sectional (A) and longitudinal (B) studies in terms of study design, autoimmune diseases, and sample size. (C) Geographic distribution of the selected studies.

## Slide 4
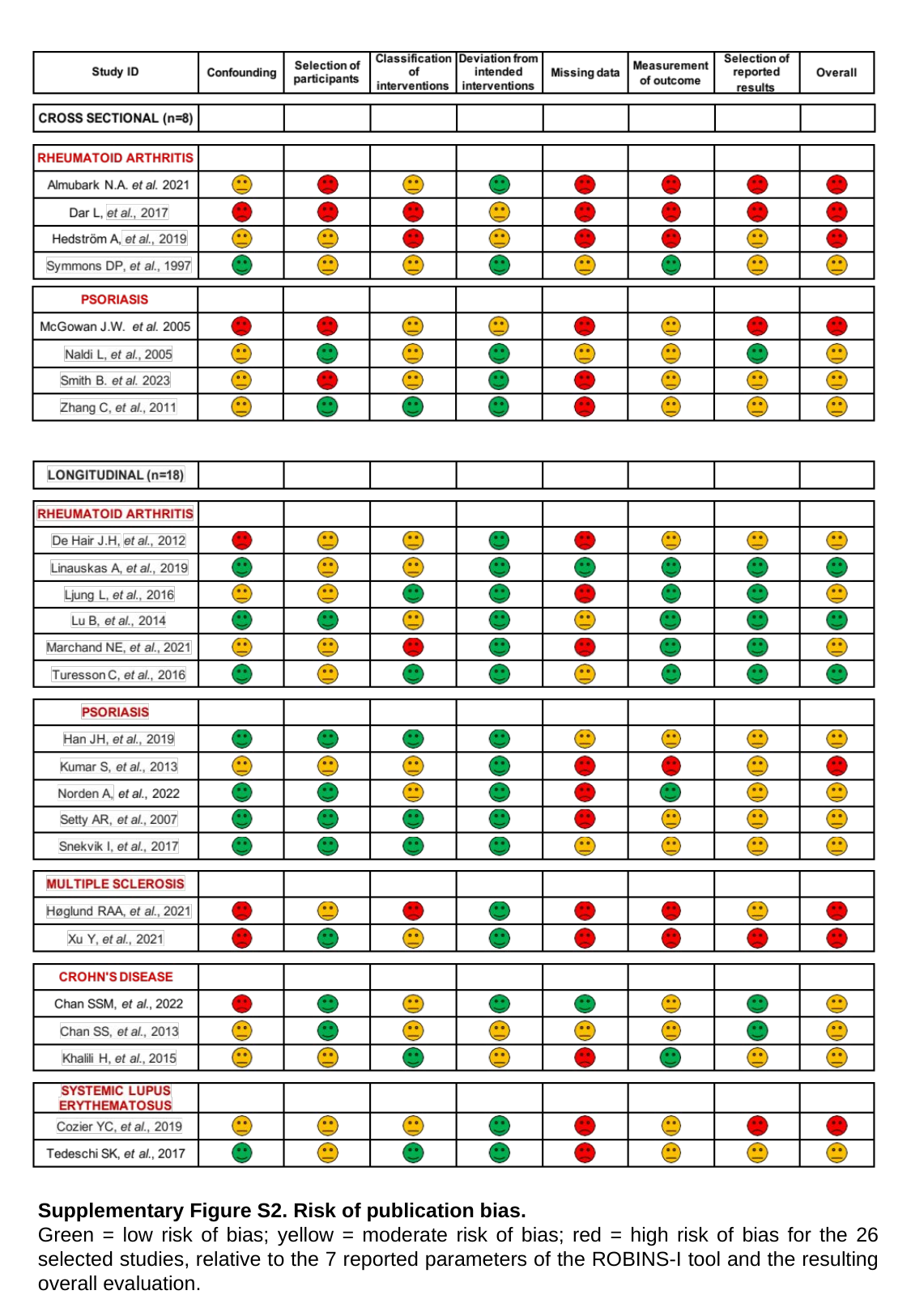

Supplementary Figure S2. Risk of publication bias.
Green = low risk of bias; yellow = moderate risk of bias; red = high risk of bias for the 26 selected studies, relative to the 7 reported parameters of the ROBINS-I tool and the resulting overall evaluation.

## Slide 5
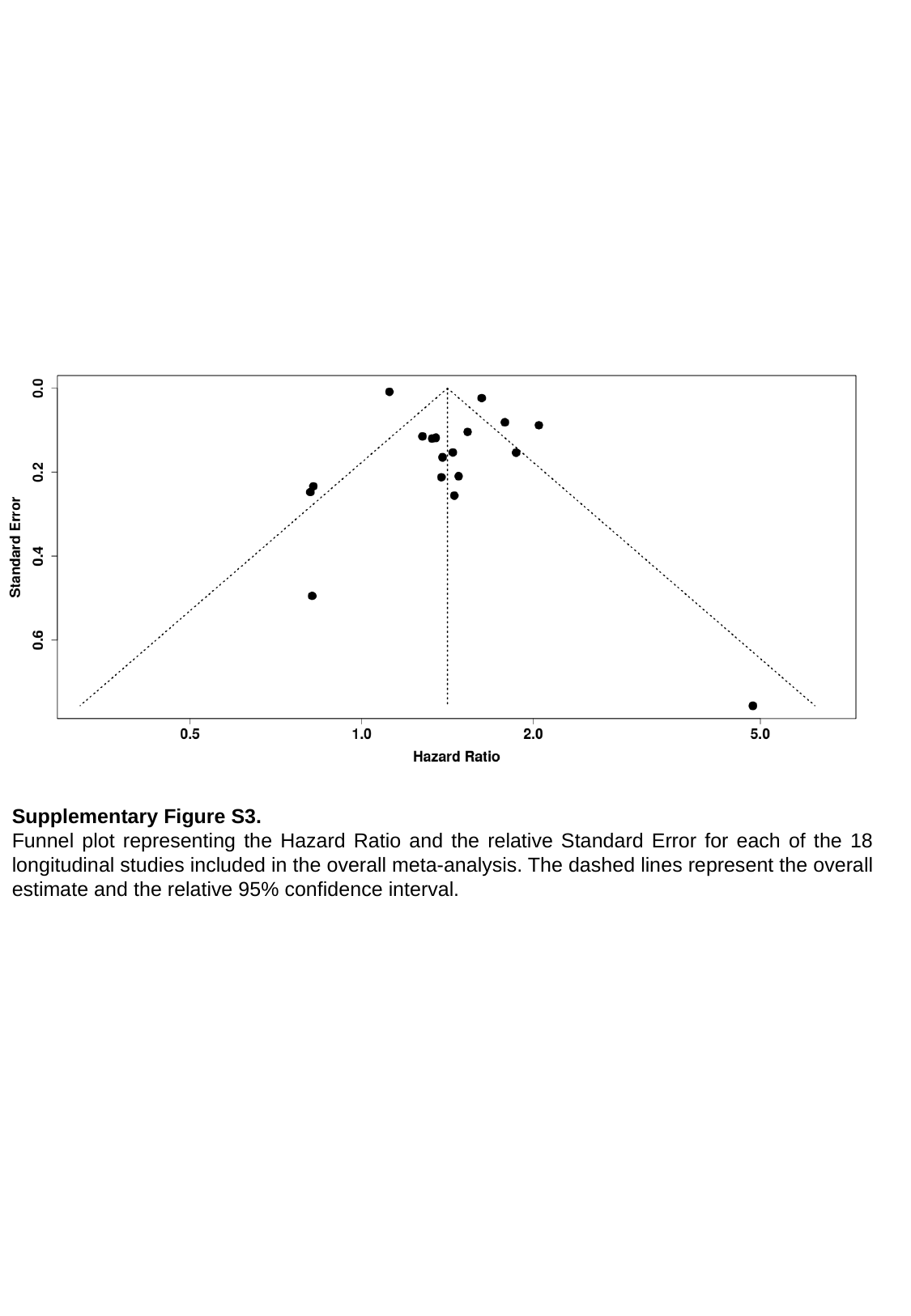

Supplementary Figure S3.
Funnel plot representing the Hazard Ratio and the relative Standard Error for each of the 18 longitudinal studies included in the overall meta-analysis. The dashed lines represent the overall estimate and the relative 95% confidence interval.
