# Supplementary material for: Obesity as a Risk Factor for Autoimmune Diseases: A Systematic Review and Meta‐Analysis
Source: Obesity (Silver Spring). 2025 Nov 4;34(1):36–50. doi: 10.1002/oby.70044 (PMC12724045; doi:10.1002/oby.70044)
Supplement: Supplementary file 2 — Table S1: References of the 35 studies potentially useful for testing the association between obesity and autoimmune diseases, and then excluded for the reasons indicated in the last column on the right (see also Figure 1). [file OBY-34-36-s001.docx]

|  | **Publication doi** | **Publication Title** | **Reasons for Exclusion** |
| --- | --- | --- | --- |
| **Autoimmune tiroiditis/Hashimoto’s Thyroiditis** |  |  |  |
| Huo J. *et al.* (2023) | 10.1186/s40001-023-01480-1 | Causal association between body mass index and autoimmune thyroiditis: evidence from Mendelian randomization | Genetic analysis |
| Amouzegar A. *et al.* (2020) | 10.1080/07435800.2020.1749847 | Abdominal Obesity Phenotypes and Incidence of Thyroid Autoimmunity: A 9-Year Follow-up | Not pertinent |
| J.Y. Yao *et al.* (2021) | 10.3967/bes2021.111 | Obesity rather than Metabolic Syndrome is a Risk Factor for Subclinical Hypothyroidism and Thyroid Autoimmunity | Not pertinent |
| Yang H. *et al.* (2021) | <https://doi.org/10.2147/JIR.S353384> | Gender-Specific Impact of Metabolic Obesity Phenotypes on the Risk of Hashimoto’s Thyroiditis: A Retrospective Data Analysis Using a Health Check-Up Database | Unavaible data for any cumulative analysis |
| **Crohn's disease and ulcerative colitis** |  |  |  |
| Sehgal P. *et al.* (2023) | 10.1136/flgastro-2022-102276 | Obesity among those newly diagnosed with Crohn's disease and ulcerative colitis compared with the general population | Not pertinent |
| Je Y. *et al.* (2023) | 10.1093/ecco-jcc/jjac193 | Association of Waist Circumference with the Risk of Inflammatory Bowel Disease: a Nationwide Cohort Study of 10 Million Individuals in Korea | Not pertinent |
| Mendall M.A, *et al.* (2011) | 10.1007/s10620-010-1541-6 | Is obesity a risk factor for Crohn's disease? | Not pertinent |
| Mendall M.A. *et al.* (2019) | 10.1038/s41598-019-42642-8 | Body mass index in young men and risk of inflammatory bowel disease through adult life: A population-based Danish cohort study | Unavaible data for any cumulative analysis |
| Mendall M. *et al.* (2018) | 10.1371/journal.pone.0190600 | Relation of body mass index to risk of developing inflammatory bowel disease amongst women in the Danish National Birth Cohort | Not pertinent |
| Losurdo G. *et al.* (2020) | 10.3748/wjg.v26.i47.7528 | Prevalence and associated factors of obesity in inflammatory bowel disease: A case-control study | Not pertinent |
| Lima J.S. *et al.* (2023) | 10.2147/CEG.S436699 | Body Mass Index Profile of Adult Patients with Inflammatory Bowel Disease in a Multicenter Study in Northeastern Brazil | Not pertinent |
| **Multiple sclerosis** |  |  |  |
| Al-Wutayd O.A. *et al.* (2022) | 10.17712/nsj.2022.2.20210121 | Association between obesity during different age periods and multiple sclerosis in Saudi Arabia: A multicenter case-control study | Not pertinent |
| Harroud A. *et al.* (2021) | 10.1177/1352458521995484 | The relative contributions of obesity, vitamin D, leptin, and adiponectin to multiple sclerosis risk: A Mendelian randomization mediation analysis | Not pertinent |
| Manouchehrinia A. et al. (2018) | 10.3389/fneur.2018.00232 | Association of Pre-Disease Body Mass Index With Multiple Sclerosis Prognosis | Not pertinent |
| Vandebergh M. *et al*. (2022) | 10.1212/NXI.0000000000001165 | Effects of Vitamin D and Body Mass Index on Disease Risk and Relapse Hazard in Multiple Sclerosis: A Mendelian Randomization Study | Not pertinent |
| Siokas V. *et al.* (2021) | 10.3390/neurolint13040051 | Impact of Body Mass Index on the Age of Relapsing-Remitting Multiple Sclerosis Onset: A Retrospective Study | Not pertinent |
| Wang X. *et al.* (2024) | 10.1038/s41598-024-57260-2 | Genetic causal role of body mass index in multiple neurological diseases | Genetic analysis |
| Zeng R. *et al.* (2023) | 10.1016/j.ebiom.2023.104647 | Dissecting shared genetic architecture between obesity and multiple sclerosis | Genetic analysis |
| Khurana S.R. *et al.* (2009) | 10.1097/PHM.0b013e318194f8b5 | The prevalence of overweight and obesity in veterans with multiple sclerosis | Not pertinent |
| Mokry L.E. *et al.* (2016) | 10.1371/journal.pmed.1002053 | Obesity and Multiple Sclerosis: A Mendelian Randomization Study | Genetic analysis |

|  | **Publication doi** | **Publication Title** | **Reasons for Exclusion** |
| --- | --- | --- | --- |
| **Psoriatic arthritis** |  |  |  |
| Li W. *et al.* (2012) | 10.1136/annrheumdis-2011-201273 | Obesity and risk of incident psoriatic arthritis in US women | Unavaible data for any cumulative analysis |
| Soltani-Arabshahi R. *et al.* (2010) | 10.1001/archdermatol.2010.141 | Obesity in early adulthood as a risk factor for psoriatic arthritis | Not pertinent |
| Queiro R. *et al.* (2019) | 10.1097/MD.0000000000016400 | Obesity in psoriatic arthritis: Comparative prevalence and associated factors | Not pertinent |
| **Psoriasis** |  |  |  |
| Dai Q. *et al.* (2023) | 10.1007/s00403-023-02780-6 | Causal effect of educational attainment on psoriasis risk mediated by obesity-related traits: Mendelian randomization study | Not pertinent |
| Rathod A, *et al.* (2022) | 10.4103/idoj.idoj_59_22 | Palmoplantar Plaque Psoriasis is Associated with Diabetes, Hypertension, Obesity, and Metabolic Syndrome-A Case-Control Study | Not pertinent |
| Herron M.D. *et al.* (2005) | 10.1001/archderm.141.12.1527. | Impact of obesity and smoking on psoriasis presentation and management | Not pertinent |
| Danielsen K. *et al.* (2017) | 10.2340/00015555-2530 | Overweight and Weight Gain Predict Psoriasis Development in a Population-based Cohort | Unavaible data for any cumulative analysis |
| **Rheumatoid arthritis** |  |  |  |
| Crowson C.S. *et al.* (2013) | 10.1002/acr.21660 | Contribution of obesity to the rise in incidence of rheumatoid arthritis | Unavaible data for any cumulative analysis |
| Karlsson T. *et al.* (2023) | 10.1002/art.42613 | Body Mass Index and the Risk of Rheumatic Disease: Linear and Nonlinear Mendelian Randomization Analyses | Unavaible data for any cumulative analysis |
| Tang B, *et al.* (2020) | 10.1002/art.41517 | Obesity-Related Traits and the Development of Rheumatoid Arthritis: Evidence From Genetic Data | Genetic analysis |
| Müller R. *et al.* (2016) | 10.1007/s00296-016-3464-9 | The metabolic profile in early rheumatoid arthritis: a high prevalence of metabolic obesity | Not pertinent |
| Fu L, et al. (2018) | 10.1080/14397595.2017.1307711 | A case-control study of rheumatoid arthritis revealed abdominal obesity and environmental risk factor interactions in northern China | Not pertinent |
| **Sjögren's disease** |  |  |  |
| McCoy S. et al. (2022) | 10.1007/s10067-022-06226-8 | Sex hormones, body mass index, and related comorbidities associated with developing Sjögren's disease: a nested case-control study (Not pertinent) | Not pertinent |
| Servioli L. *et al.* (2019) | 10.3899/jrheum.180481 | Association of Smoking and Obesity on the Risk of Developing Primary Sjögren Syndrome: A Population-based Cohort Study | Unavaible data for any cumulative analysis |
| **Systemic lupus erythematosus** |  |  |  |
| Versini M. *et al.* (2017) | 10.1111/eci.12757. | Smoking and obesity in systemic lupus erythematosus: a cross-sectional study | Unavaible data for any cumulative analysis |

**Table S1.** References of the 35 studies potentially useful for testing the association between obesity and autoimmune diseases, and then excluded for the reasons indicated in the last column on the right (see also Figure 1).
